# Spring-Induced Mechanical Strategy for High-Output, Flexible PAN-Based Piezoelectric Harvester

**DOI:** 10.3390/ma19051039

**Published:** 2026-03-09

**Authors:** Quan Hu, Yueyue Yu, Ru Guo, Hang Luo

**Affiliations:** State Key Laboratory of Powder Metallurgy, Powder Metallurgy Research Institute, Central South University, Changsha 410083, China; huquan@csu.edu.cn (Q.H.); 15090568095@163.com (Y.Y.); hangluo@csu.edu.cn (H.L.)

**Keywords:** piezoelectric nanogenerators, spring-induced mechanical strategy, output performance, peak power density, polyacrylonitrile–barium titanate nanocomposite

## Abstract

The growing demand for wearable electronics and the Internet of Things (IoT) calls for flexible piezoelectric energy harvesters with substantially improved power output. Polyacrylonitrile (PAN) polymers, with their high polarization and excellent thermal stability, are among the most promising candidates for efficient flexible piezoelectric materials. However, the performance of existing PAN-based harvesters remains limited, and strategies for further enhancing their output are still insufficiently explored. Herein, this study aims to overcome the output bottleneck of PAN-based PENGs by implementing a novel mechanical excitation strategy. Using electrospun flexible PAN-BaTiO_3_ nanocomposite films, we systematically compared the electromechanical responses under conventional compression and impact modes. Real-time synchronized force–current measurements in compression mode revealed that the output current increases progressively with drive frequency (2–10 Hz). Specifically, the PENG with PAN-20 wt.% BaTiO_3_ achieved a peak current of 0.33 mA at 10 Hz, showing an approximately 7.9-fold enhancement over its pure PAN counterpart. More importantly, under 6 Hz impact excitation, the device exhibited a remarkable output current density of 1.0 mA cm^−2^ and a peak power density of 256.5 µW cm^−2^. This current density is 95 times higher than that in compression mode at a comparable frequency and surpasses the performance of most recently reported piezoelectric and triboelectric nanogenerators. With an effective area of 16 cm^2^, the PENG could simultaneously illuminate up to 275 commercial LEDs or 100 individual bulbs and maintained stable operation over 63,530 cycles. This work overcomes the output bottleneck in low-frequency energy harvesting and provides an effective pathway toward practical energy-harvesting applications.

## 1. Introduction

Amidst the urgent global imperative for decarbonization and energy transition, the development of sustainable energy technologies has become increasingly paramount [1]. Driven by the Internet of Things and artificial intelligence in particular, the sustained power supply for vast numbers of distributed sensing nodes and wearable devices has emerged as a critical bottleneck constraining their advancement [2,3]. Against this backdrop, energy harvesting technologies capable of collecting and converting dispersed mechanical energy from the environment offer a highly promising solution pathway for constructing self-powered, low-maintenance microelectronic systems. Among these, piezoelectric nanogenerators (PENGs) are particularly distinguished by their high electromechanical conversion efficiency, robust environmental stability, and ease of miniaturization and integration. However, their relatively low power density remains the core bottleneck constraining the technology’s transition to large-scale practical application [4,5].

PENG leverages the piezoelectric effect to convert mechanical energy into electrical energy [6]. To address the limitations in output capacity, recent research has prioritized enhancing output current and power density through material innovation, structural optimization, and interface engineering [7,8]. A prominent approach involves the development of piezoelectric composites, which achieve an ingenious synergy between high-performance inorganic materials and flexible organic polymers [9]. This hybrid strategy effectively extends the performance limits of PENGs [10]. For instance, BaTiO_3_ (BTO) nanofillers are incorporated into non-piezoelectric polymers such as resins [11], PDMS [12,13] or PVC [14] to enhance flexibility, or integrated with piezoelectric polymers like PVDF [15], P(VDF-HFP) [16], P(VDF-TrFE) [17], or polylactic acid (PLA) [18] to improve piezoelectric performance. Specifically, Cho employed a phase-separated nanocoating method to fabricate highly oriented BaTiO_3_@P(VDF-TrFE) nanocomposites, thereby enhancing the piezoelectric performance for applications in three-dimensional piezoelectric films [19]. Guan anchored poly(dopamine)-modified barium titanate nanoparticles onto a P(VDF-TrFE) fiber surface, creating a hierarchical microstructure that significantly elevated the PENG’s output voltage [20].

Recently, polyacrylonitrile (PAN) has attracted considerable attention as a highly promising piezoelectric polymer. This interest is driven by the strong dipole moment of its -CN groups (3.5 Debye) and intensive hydrogen bonding, which collectively endow PAN with superior mechanical and piezoelectric characteristics. A key advantage of PAN is its ability to form a thermally stable trapezoidal structure through controlled heat treatment without losing dipole orientation, making it suitable for high-temperature applications [21,22]. Electrospinning has become a pivotal technique for fabricating nanofibrous materials with tailorable morphologies and properties, offering vast potential in energy harvesting, biomedicine, and sensing [23,24]. Recent studies have focused on further elevating the output of these PAN-based PENGs. Wang highlighted that PAN nanofibers can surpass the performance of conventional PVDF, achieving a 2.0 V output from a small-scale (5 cm^2^) device [25]. However, these enhancement strategies often necessitate intricate material synthesis or structural designs. More facile and effective strategies to boost the output of PAN-based PENGs remain highly sought after.

From a fundamental energy conversion perspective, the mechanical excitation mode is a critical determinant of electromechanical response efficiency. However, compared to the extensive efforts dedicated to material and structural engineering, the systematic optimization of excitation modes remains relatively unexplored, leaving their full performance potential untapped [26]. Herein, we apply this spring impact strategy to PAN-BaTiO_3_ nanofiber-based PENGs to systematically validate its material versatility and to overcome the low-frequency output bottleneck [27]. Our results demonstrate that the PENG incorporating 20 wt.% BaTiO_3_ achieves a peak current of 0.33 mA under 10 Hz compression, representing an approximately 7.9-fold enhancement compared to pure PAN devices. Further, under a 6 Hz impact excitation mode, its output current density and peak power density reached 1 mA cm^−2^ and 256.5 mW cm^−2^, respectively, surpassing the majority of recently reported piezoelectric and triboelectric nanogenerators. Furthermore, with an effective area of 16 cm^2^, this PENG can easily power up to 275 LEDs or 100 individual light bulbs, maintaining excellent operational stability over 63,530 cycles. These findings confirm the broad applicability of this excitation strategy, providing a robust approach for optimizing PAN polymer-based piezoelectric harvesting systems and advancing their practical utility in low-frequency environments.

## 2. Materials and Methods

### 2.1. Materials

N,N-dimethylformamide (DMF, 98% purity), polyacrylonitrile (PAN, 99% purity), and BaTiO_3_ nanoparticle powder (99% purity) were all obtained from Sigma Aldrich (St. Louis, MO, USA) and were not subjected to further processing.

### 2.2. Preparation of PAN-BaTiO_3_ Nanofibre Film

To prepare the precursor solutions, varying amounts of BaTiO_3_ nanoparticles (0 g, 0.353 g, 0.706 g, and 1.059 g) were dispersed in 20 g of DMF via ultrasonication. Subsequently, 3.53 g of PAN powder was added to each dispersion and stirred at 60 °C for 6 h to ensure complete dissolution. This yielded four distinct spinning solutions: pure PAN, PAN-10 wt.% BaTiO_3_, PAN-20 wt.% BaTiO_3_, and PAN-30 wt.% BaTiO_3_, all maintained at a polymer concentration of 15 wt.%. The electrospinning process was conducted under uniform parameters: a 23 G needle, a needle voltage of 4 kV, a collector (drum) voltage of 10 kV, a rotation speed of 1000 rpm, a flow rate of 1 mL/h, and a tip-to-collector distance of 10 cm.

### 2.3. Preparation of Piezoelectric Nanogenerator (PENG) Devices

**Figure 1:**
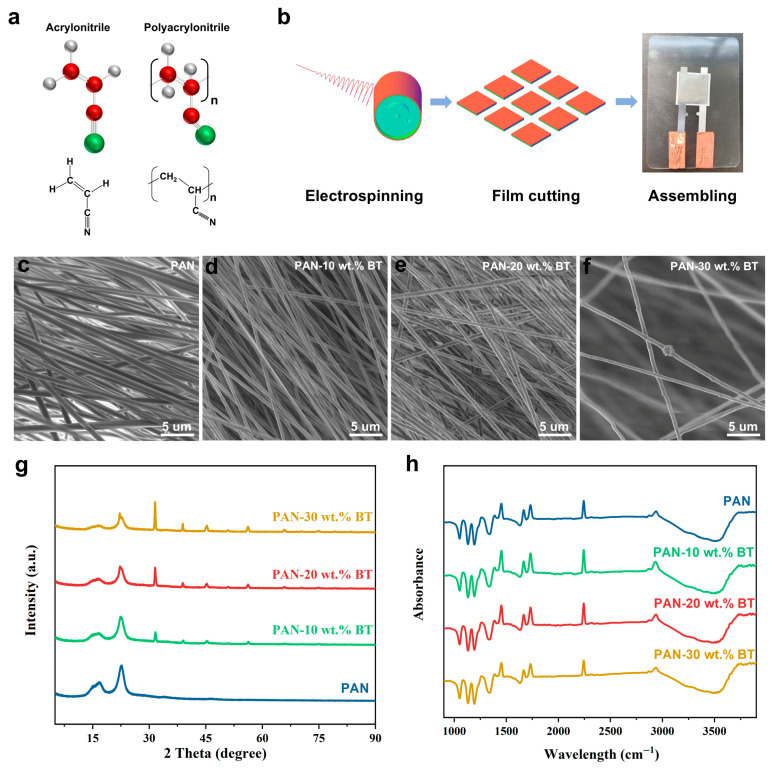
(**a**) Schematic diagrams of the structures of acrylonitrile and polyacrylonitrile. (**b**) Schematic diagram of the fabrication process of electrospun PAN-BaTiO_3_ nanofiber film and PENG device. (**c**–**f**) SEM images of electrospun PAN-BaTiO_3_ nanofiber film with different BaTiO_3_ filler contents. (**g**) XRD patterns and (**h**) FTIR spectrum of electrospun PAN-BaTiO_3_ nanofiber film.

PENG devices were fabricated using a layer-by-layer encapsulation strategy. The as-prepared PAN-BaTiO_3_ nanofiber membranes were first tailored to the appropriate size with aluminum electrodes affixed to both sides. To establish electrical connections, copper strips were attached to the electrode surfaces as test leads. The entire assembly was then sandwiched between PET films and sealed via hot pressing at 100 °C for 10 s. Finally, the device was thoroughly compacted to eliminate internal air, resulting in a robustly encapsulated sandwich-structured PENG, as schematically illustrated in Figure 1b.

### 2.4. Characterization

The morphology of electrospun PAN-BaTiO_3_ nanofiber film was characterized by a field-emission scanning electron microscope (SEM, Nova, Nano SEM, 450, Hillsboro, OR, USA). Phase and structural analyses were performed using X-ray diffraction (XRD, Rigaku D/MAX 2500 diffractometer (Tokyo, Japan) equipped with a Cu Kα source) in the 2θ range of 10–70° and Fourier-transform infrared spectroscopy (FTIR, Thermo Scientific Nicolet iS10, Waltham, MA, USA) in the wavenumber range of 400–4000 cm^−1^ to identify the functional groups. Mechanical excitation was applied in two modes: (i) Compression mode: a linear stepper motor applied a cyclic force with controlled frequency (2–10 Hz). (ii) Impact mode: three stainless steel springs with different stiffness coefficients (k = 45 N/m, 96 N/m, and 205 N/m) were used with an impact stroke of 10 mm, velocity of ~0.89 m·s^−1^, and block mass of 53 g. The spring was compressed and released to propel a metal block to impact the PENG, creating a transient force. These stiffness coefficients values were selected to investigate the effect of impact intensity and duration on the electromechanical response of the PENG, as a stiffer spring delivers a higher force in a shorter time. The applied force and the output current/voltage of the PENG were recorded using a mechanical sensor and a digital multimeter (Keithley DMM7510, Shanghai, China), respectively. All measurements were synchronized for accurate correlation between mechanical input and electrical output.

## 3. Results and Discussion

As shown in Figure 1a, although the acrylonitrile monomer has a strong molecular dipole moment owing to its cyano group, its disordered arrangement results in macroscopic isotropic and central symmetry, leading to a lack of piezoelectricity. Conventionally synthesized polyacrylonitrile (PAN) typically exhibits a random stereostructure with chaotically oriented cyano side chains. While strong dipole interactions exist locally, they cancel each other out macroscopically, rendering the material non-piezoelectric overall [28,29]. Electrospinning can break this symmetry, enabling the orientation of dipoles. Specifically, under a high-voltage electric field, the extreme stretching and electrostatic forces during fiber formation promote the orientation of dipoles, endowing the resulting PAN nanofiber membrane with an intrinsic piezoelectric response [25,30,31]. To construct a high-performance piezoelectric nanogenerator (PENG), the electrospun PAN-BaTiO_3_ nanofiber membranes were assembled into a typical sandwich-structured device (See Section 2.3 for details). The microstructure of PAN-BaTiO_3_ nanofiber membranes was characterized using scanning electron microscopy (SEM). As shown in Figure 1c–f, the electrospun fibers exhibit a uniform, bead-free morphology with diameters primarily distributed between 500 and 700 nm. With increasing BT doping concentration, more BaTiO_3_ nanoparticles are observed to be uniformly embedded within the PAN fiber matrix, though at 30 wt.%, slight agglomeration can be observed on the fiber surface. The phase composition of the composite films was analyzed via X-ray diffraction (XRD) (Figure 1g). Compared to the diffraction pattern of the pure PAN film, all composite films exhibited new diffraction peaks at approximately 2θ = 31.5°, 38.9°, 45.4°, and 56.3°, corresponding to the (101), (111), (200), and (211) crystal planes of tetragonal BaTiO_3_ (JCPDS card no.: 05-0626), and confirming the successful BaTiO_3_ nanoparticle incorporation. The intensity of these peaks showed a clear increasing trend with higher BaTiO_3_ loading. FT-IR spectroscopy (Figure 1h) revealed the functional group of the PAN-based composite film. The characteristic -C≡N stretch of PAN at ~2242 cm^−1^ [32], along with CH_2_ vibrations at 2935 cm^−1^ and 1452 cm^−1^, were observed in all composites [33]. The FTIR spectra primarily show the characteristic peaks of the PAN matrix. The characteristic Ti-O vibrational modes of BaTiO_3_, typically found in the far-infrared region (<800 cm^−1^), are not distinctly observed here, likely due to the measurement range and the strong background absorption of the polymer matrix. A closer inspection of the -C=N peak at ~2242 cm^−1^ reveals a slight broadening and a minor shift to a lower wavenumber in the composite samples, particularly at 20 wt.% loading, which may suggest a weak dipolar interaction between the -CN groups of PAN and the surface of the BaTiO_3_ nanoparticles.

**Figure 2:**
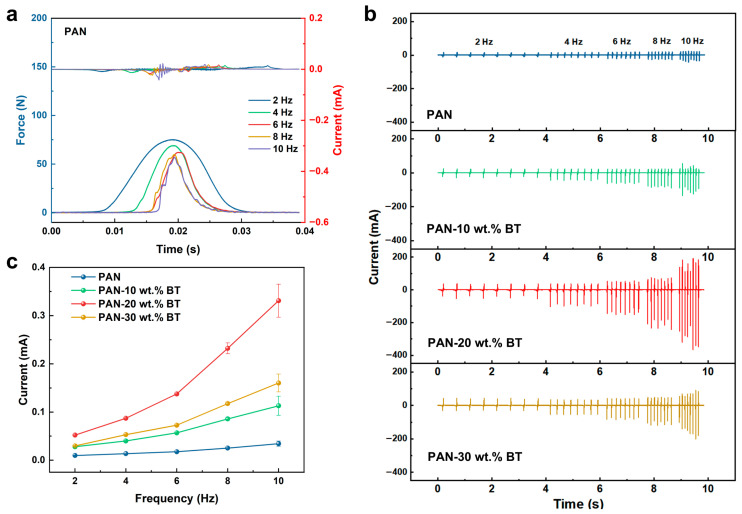
(**a**) The enlarged force and current curve within one cycle. (**b**) Applied force and output current of PAN-based PENG under compression model with increasing driving frequencies. (**c**) Output current of PAN-BaTiO_3_ composite film with different filler content.

The piezoelectric output performance of PAN-BaTiO_3_ composite films (2 cm × 2 cm) was first evaluated under conventional compression mode. During testing, a linear motor generated periodic mechanical motion to apply controlled pressure to the film. The resulting piezoelectric signals were acquired by a high-precision electrometer, while a force sensor synchronously monitored the applied force in real time, enabling precise correlation between the electrical output and mechanical input. To elucidate the frequency dependence of the output characteristics, Figure 2a compares the applied force curve and the corresponding output current within one motion cycle, illustrating the frequency-dependent characteristics. During the compression and release, the piezoelectric output exhibits a pair of current peaks with opposite polarities. The measured force peak value exhibits a slight decay from approximately 75 N to 56 N. This phenomenon is partially attributable to a reduction in the efficiency of mechanical energy transfer at the interfaces and a diminished strain response of the viscoelastic polymer matrix under the higher driving frequencies used in this study (up to 10 Hz) [27]. This study systematically investigated the phenomenon where the output characteristics of PENG are in different drive frequencies. As shown in Figure 2b, the output current of the pure PAN-based PENG increased markedly from 0.015 mA to 0.042 mA as the frequency rose from 2 Hz to 10 Hz. This phenomenon is attributed to the fundamental nature of current as the rate of charge flow (I = d_Q_/d_t_). At higher driving frequencies, the mechanical deformation and subsequent charge generation and transfer occur within a shorter time interval (Δt), leading to a higher instantaneous peak current, even if the total generated charge (Q) per cycle remains similar. Furthermore, the output performance of PAN-based composites with varying BaTiO_3_ content was compared (Figure 2c). As the frequency increases from 2 Hz to 10 Hz, the output current of the composite film-based PENG gradually rises. Under the same frequency, the output current of the composite material exhibits an initial increase followed by a decrease with rising BaTiO_3_ content. Among all samples, the PAN-BaTiO_3_ composite film containing 20 wt.% BaTiO_3_ demonstrated the highest peak output current (0.33 mA), representing a 7.9-fold enhancement compared to the PENG derived from pure PAN (0.042 mA). While further increasing the frequency promises even greater performance, practical application faces two key challenges. First, ambient vibration energy is predominantly concentrated in low-frequency ranges (typically below 10 Hz), which mismatches the natural resonance frequencies of most piezoelectric harvesters (nearly in the kilohertz range for PAN thin-film devices). Second, conventional compress excitation methods using linear motors are technically limited in achieving higher frequencies. Therefore, developing novel mechanical excitation strategies that can substantially boost the current output within applicable low-frequency ranges is of significant research value and practical importance.

**Figure 3:**
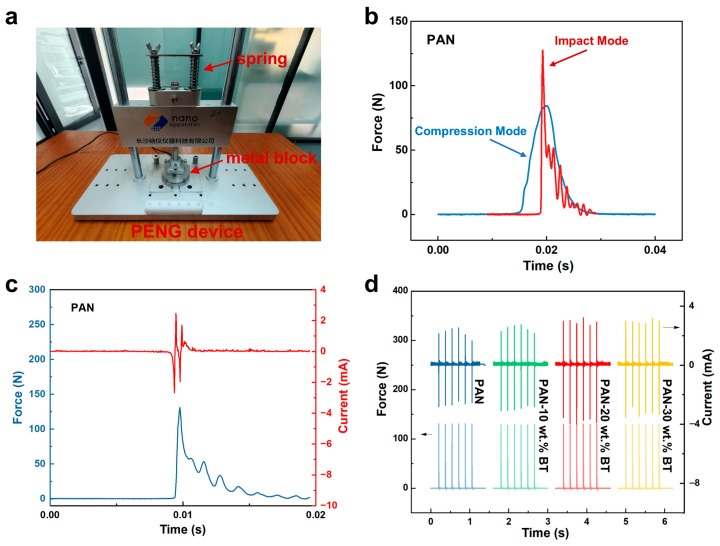
Piezoelectric output performance under impact mode. (**a**) Schematic diagram of the device system. (**b**) The applied force curve under impact mode and compression mode under driving frequencies of 6 Hz. (**c**) The enlarged force and output current curve within one cycle. (**d**) Output current of PAN-BaTiO_3_ composite film with different BT contents.

Inspired by the energy release characteristic of crossbows that converts low-frequency motion into high-frequency vibrations, we employ a recently proposed simplified and efficient strategy based on transient shocks to maximize piezoelectric output current. As illustrated in Figure 3a, this strategy employs a spring mechanism at the mechanical loading end to apply transient stress to the piezoelectric device by rapidly propelling a weight. The piezoelectric output generated under such transient mechanical excitation is defined as the impact mode [27]. To simulate low-frequency vibration energy in practical environments, the drive frequency for all impact modes is maintained at 6 Hz. In impact mode, the applied force features a short duration and concentrated distribution, enabling PENGs to generate significantly higher currents at low-frequency range. Compared to the conventional compression mode, the force magnitude applied in impact mode exhibits pronounced multimodal fluctuations (Figure 3b). As shown in Figure 3c, a single impact loading cycle triggers multiple excitation force pulses on the piezoelectric device, each of which produces a corresponding piezoelectric current signal. Both the impact force and the initial output current pulse reach their peak values simultaneously, with subsequent pulses decaying progressively due to the continuous dissipation of mechanical energy. Notably, although the peak force in impact mode (130 N) is only marginally higher than that in conventional compression mode (100 N), the output current of the pure PAN-based piezoelectric nanogenerator (2 × 2 cm^2^) under impact achieves a remarkably high output current of 2.7 mA. This enhancement primarily stems from the marked reduction in full-width at the half maximum (FWHM) of response time during impact mode, thereby increasing the charge transfer rate per unit time. Further impact loading was applied to PENG composites with varying BaTiO_3_ contents. As shown in Figure 3d, the output current progressively increased with rising BaTiO_3_ filler content, reaching a maximum of 3.99 mA in the PAN-20 wt.% BaTiO_3_ nanocomposite. This variation stems from the reason that the introduction of highly rigid BaTiO_3_ particles into the PAN matrix enhances the mechanical quality factor (Q_m_) of the composite film. Q_m_ is a dimensionless parameter that describes the sharpness of resonance and the efficiency of a material to store mechanical energy versus dissipating it. A higher Q_m_ results in more pronounced oscillatory behavior under impulsive loading, thereby facilitating stronger mechanical resonance excitation upon impact, explaining the enhanced output.

**Figure 4:**
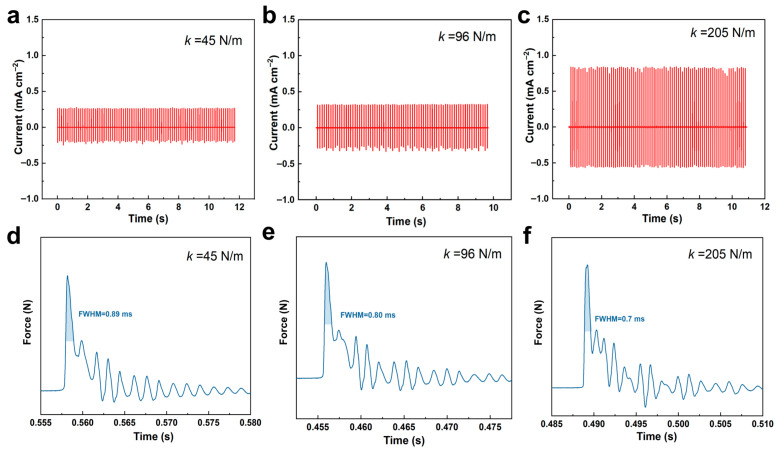
Influence of spring stiffness coefficients on the mechanical and electrical responses in impact mode: (**a**–**c**) Loading force and (**d**–**f**) corresponding output current at stiffness values of 45 N/m, 96 N/m, and 205 N/m, respectively.

The influence of a spring with different stiffness coefficients on output performance under impact mode has been systematically investigated. As illustrated in Figure 4, increasing the stiffness coefficient (*k*) leads to a significant rise in the PENG’s output current, accompanied by a corresponding reduction in the duration of the applied mechanical force (i.e., a decrease in the full width at half maximum (FWHM) of the force curve). As is known, the theoretical short-circuit current (*I*_sc_) is expressed as the amount of transferred charges (*Q*) per unit time through electric circuit:

$$I_{sc}=\frac{Q}{t}=\frac{d_{33}}{\Delta t}F$$

where *d*_33_ denotes the piezoelectric charge coefficient and *F* represents the applied force. Since the generated charge is proportional to the applied stress, a reduction in the response time (Δt), which is the interval between force application and charge transfer, results in a significant enhancement of the peak current. This analysis identifies the duration of force application as a critical determinant of piezoelectric performance. Consequently, increasing the spring stiffness coefficient can effectively boost the output current by minimizing the delay in the electromechanical response. The charge quantity is proportional to the applied stress. An increase in the material’s elastic modulus reduces the response time (Δt) between external force application and charge transfer, thereby significantly enhancing the observed peak current. The aforementioned analysis indicates that the duration of force application is one of the key factors determining piezoelectric output performance. Consequently, appropriately enhancing the material’s elastic modulus can effectively boost output current by reducing the delay in the mechanical-to-electrical response.

To further evaluate the output voltage characteristics of PENG devices under impact mode, an external variable resistor method was employed for quantitative assessment. Based on the equivalent circuit model

$$I=U/\left(R+r\right)$$

**Figure 5:**
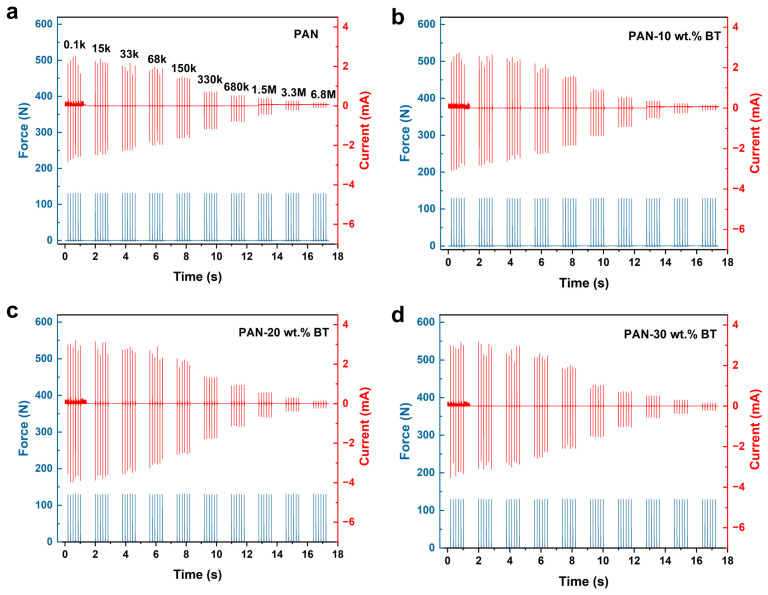
In impact mode, the output current of (**a**) PAN, (**b**) PAN-10 wt.% BT, (**c**) PAN-20 wt.% BT, and (**d**) PAN-30 wt.% BT-based PENG varies with changes in external impedance (from 0.1 kΩ to 6.8 MΩ).

**Figure 6:**
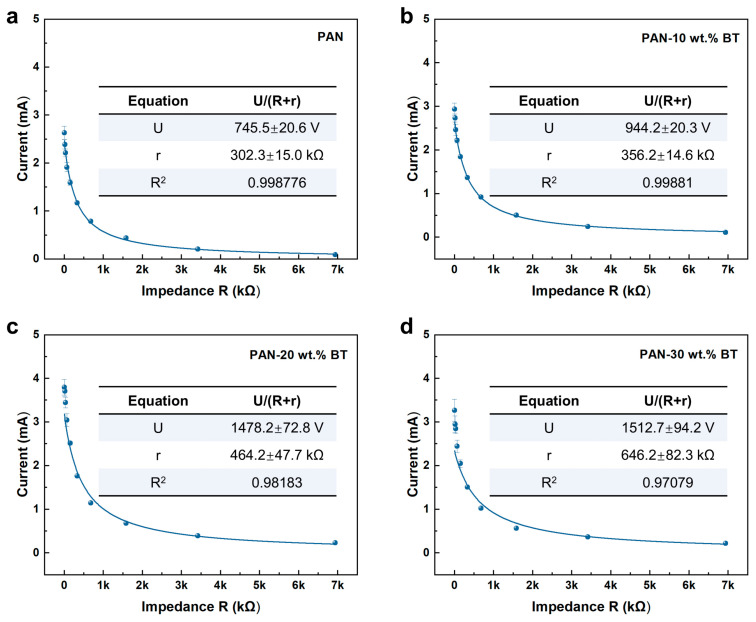
The open-circuit voltage and internal resistance of the (**a**) PAN, (**b**) PAN-10 wt.% BT, (**c**) PAN-20 wt.% BT, and (**d**) PAN-30 wt.% BT-based PENG were determined by fitting the current-load resistance (*I*-*R*) relationship curves.

The open-circuit voltage *V*_oc_ and internal resistance *r* were determined by fitting the output current *I* measured across a range of precision variable external load resistors R (ranging from 0.1 kΩ to 6.8 MΩ). As shown in Figure 5 and Figure 6 and Table 1, the fitted parameters for the pure PAN-based PENG yielded a *V*_oc_ of 745.5 V and an internal resistance *r* of 302.3 kΩ, with a coefficient of determination (R^2^) of 99.8776%. For PAN nanocomposites containing BaTiO_3_ at 10 wt.%, 20 wt.%, and 30 wt.%, the open-circuit voltages and corresponding internal resistances were successively: 944.2 V (356.2 kΩ), 1478.2 V (464.2 kΩ), and 1512.7 V (646.2 kΩ), respectively. It is noteworthy that the fitted open-circuit voltage of up to ~1500 V approaches the estimated breakdown voltage range for nanofiber composite films of this thickness, which is typically on the order of several kilovolts based on their dielectric strength [34]. This result confirms that the impact mode can effectively drive the material to operate near its fundamental physical limits [27].

**Figure 7:**
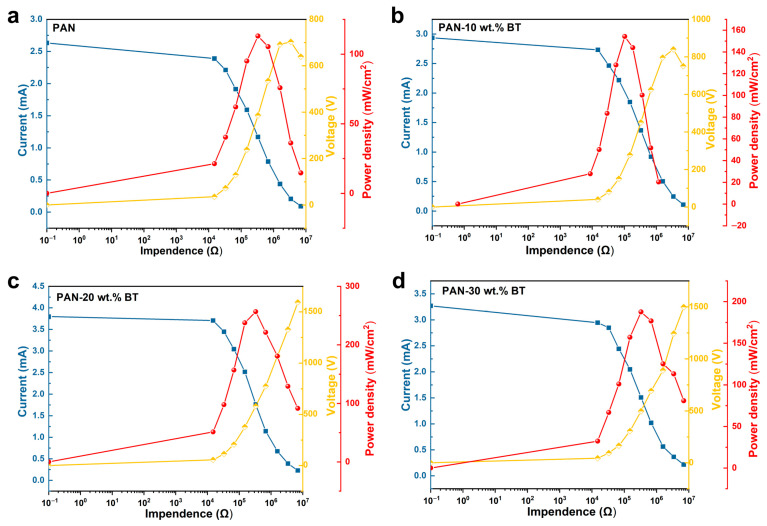
Output current and power density versus external load resistance for PENG based on (**a**) PAN, (**b**) PAN-10 wt.% BT, (**c**) PAN-20 wt.% BT, and (**d**) PAN-30 wt.% BT in impact mode.

To evaluate the output power, we employed the external load resistor method. The PENG is connected in series with a variable resistor, forming a closed circuit. By measuring the voltage drop across (or current through) this known resistance, the output power (P = I^2^R) can be calculated. As shown in Figure 7, the output voltage increases with rising load resistance, while the output current decreases correspondingly. In accordance with the impedance matching principle, the output power follows a single-peak distribution, reaching its maximum at a specific resistance value. Specifically, at a matched resistance of 330 kΩ, the PAN-20 wt.% BaTiO_3_ nanocomposite-based PENG achieves a maximum output peak power density of 256.5 mW cm^−2^ in impact mode. In this mode, the rapid transient transfer and release of piezoelectric charges effectively reduce the device’s equivalent internal resistance, thereby optimizing impedance matching and enhancing energy conversion efficiency. These superior output characteristics underscore the device’s significant potential for the high-efficiency harvesting and conversion of ambient mechanical energy.

**Figure 8:**
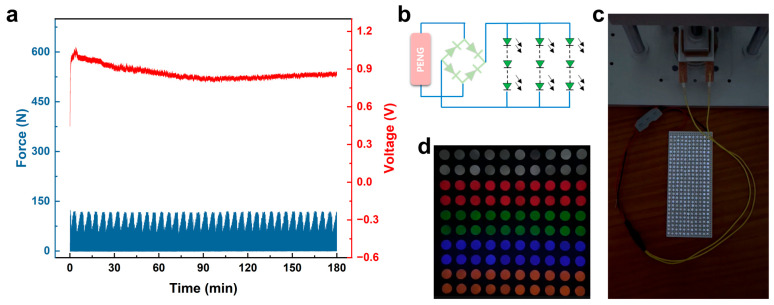
(**a**) Long-term stability of PENG operated under impact mode for 180 min. (**b**) The circuit schematic diagram of LEDs array. (**c**) Demonstration of PENG to light up 275 LEDs and (**d**) 100 light bulbs.

To evaluate the long-term operational stability of the PENG under impact conditions, continuous cyclic endurance testing was conducted. Over a cumulative period of 180 min, comprising 63,530 consecutive impact cycles, the applied excitation force remained remarkably consistent. As illustrated in Figure 8a, the charging voltage across the capacitor terminals consistently maintained between 0.82 and 1 V throughout this period, with no discernible attenuation observed. This demonstrates that the PENG device possesses outstanding reliability and operational stability. To validate its practical potential for energy harvesting, this study constructed a composite PENG device with an effective working area of 16 cm^2^, successfully driving a commercial LED array. To power the LED arrays, the AC output from the PENG was first rectified to DC using a full-wave bridge rectifier circuit (Figure 8b). A higher-resolution image has been substituted for Figure 8c. Test results demonstrate that the device can stably illuminate and sustain the normal operation of a total of 275 LEDs (Video S1, Supporting Information) and 100 light bulbs (Video S2, Supporting Information). This driving capability represents a high level of performance among current PENG research using similar materials and comparable device dimensions (Figure 8c,d).

This study conducted a comparative analysis to evaluate performance of the fabricated PENGs. The results reveal that the PENG based on the PAN-20 wt.% BaTiO_3_ composite film achieves an exceptional output current density up to 1 mA cm^−2^ and a peak power density of 256.5 mW cm^−2^ under impact excitation mode. This output current density represents a 1.5-fold increase over the pure PAN-based PENG (0.68 mA cm^−2^). Notably, the output current density under impact mode represents a 95-fold enhancement over that of the conventional compression mode, which was merely 0.0105 mA cm^−2^. Furthermore, a comprehensive performance benchmarking against recently reported piezoelectric (PENG) and triboelectric nanogenerators (TENG) is present in Table 2. Compared to conventional PENGs operating in compression mode, such as PAN/ZnO (0.15 µA/cm^2^) [35] and ZIf-8@PAN (0.28 µA/cm^2^) [36], our PAN-BaTiO_3_ device under impact mode achieves a current density over three orders of magnitude higher. Even when compared to state-of-the-art PENGs like PZT thin films (150 µA/cm^2^) [34], our device demonstrates a competitive performance of 1000 µA/cm^2^. More importantly, its peak power density of 256.5 mW/cm^2^ surpasses most other PENGs and TENGs listed, underscoring the effectiveness of our spring impact strategy.

## 4. Conclusions

In summary, this study demonstrates a significant enhancement in the output performance of PAN-based PENGs by implementing a novel mechanical excitation strategy characterized by spring-induced impact loading. Utilizing electrospun flexible PAN-BaTiO_3_ nanocomposite films, we systematically investigated the electromechanical response under both conventional compression and impact modes. Through real-time synchronized force–current curves, we first investigated the enhancement effect of increased frequency on output current in conventional compression mode. The results from real-time synchronized force–current characterization reveal that in compression mode, the output current increases progressively with drive frequency from 2 Hz to 10 Hz. Specifically, at 10 Hz, the PAN-20 wt.% BaTiO_3_ composite PENG achieves a peak current of 0.33 mA, representing an 8-fold improvement over pure PAN devices. More importantly, under 6 Hz impact excitation, the device exhibits an exceptional output current density of 1 mA cm^−2^ and a peak power density of 256.5 mW cm^−2^, respectively. This current density is 95 times higher than that achieved in conventional compression mode at a comparable frequency, surpassing the performance of most recently reported piezoelectric nanogenerators and triboelectric nanogenerators. With an effective area of 16 cm^2^, this PENG can simultaneously power up to 275 LEDs or 100 light bulbs, demonstrating excellent operational stability over 63,530 cycles. Beyond powering LEDs, the high output power density and stability of these PENGs make them promising candidates for a wide range of practical applications, including self-powered wireless sensor nodes for IoT, structural health monitoring, biomedical devices, and sustainable power sources for low-power portable electronics. This study achieved optimization of PENG output performance through a universal mechanical excitation strategy, thereby providing an effective pathway for the practical application of low-frequency energy harvesting technology.

## Figures and Tables

**Table 1:** Open-circuit voltage and internal resistance of PAN-based PENGs under impact mode, obtained from fitting the I-R curves in Figure 6.

Sample	Voc (V)	Internal Resistance, r (kΩ)	R^2^ (%)
Pure PAN	745.5	302.3	99.88
PAN-10 wt.% BT	944.2	356.2	99.88
PAN-20 wt.% BT	1478.2	464.2	98.18
PAN-30 wt.% BT	1512.7	646.2	97.08

**Table 2:** Comparison of this work with other recently reported piezoelectric and triboelectric nanogenerators.

	Materials System	Effective Size (cm^2^)	Freq.	Current Density (μA cm^−2^)	Power Density (mW cm^−2^)	Ref.
PENG	PZT thin film	3.5 × 3.5	-	150	17.5	[37]
	KNNS-BNZ-Fe ceramics	0.3 × 0.3	1 MHz	177	11.6	[38]
	PZT	-	2 Hz	279	19.1	[35]
	PAN/ZnO	4 × 4	2 Hz	0.15	1.08 × 10^−3^	[39]
	PZT/P(VDF-TrFE)	2 × 2.5	40 Hz	24	0.75	[36]
	ZIf-8@PAN/TBAHP-NMs	3 × 3	3 Hz	0.28	0.0123	[40]
	BaTiO_3_/PVDF	2 × 2	6 Hz	1040	322.2	[27]
TENG	MoS_2_@PAN	2 × 2	-	1.28	17.5	[41]
	BaTiO_3_/PVDF	-	-	1.9	0.25	[42]
	PAN/B(OH)_3_	3 × 3	-	5	66.7	[43]
	Graphene/ PVDF	-	-	18.9	13.02	[44]
	PAN/PANi	2.5 × 2.5	-	32	233	[45]
Hybrid PENG + TENG	Cs_2_Bi_2_Br_9_/ PVDF	2 × 2	5 Hz	1.63	0.234	[46]
	Spider silk/PET/ PVDF	2.5 × 2.5	-	11.52	0.4016	[47]
PENG	PAN-BaTiO_3_	2 × 2	6 Hz	1000	256.5	This work

## Data Availability

The original contributions presented in this study are included in the article/Supplementary Material. Further inquiries can be directed to the corresponding author.

## Supplementary Materials

The following supporting information can be downloaded at: https://www.mdpi.com/article/10.3390/ma19051039/s1, Video S1: PAN-BaTiO_3_ based PENG lights up 275 LEDs; Video S2: PAN-BaTiO_3_ based PENG lights up 100 bulbs.
